# Contributing roles of depression, anxiety, and impulsivity dimensions in eating behaviors styles in surgery candidates

**DOI:** 10.1186/s40337-021-00503-8

**Published:** 2021-11-08

**Authors:** Farid Benzerouk, Monique Guénin, Fabien Gierski, Delphine Raucher-Chéné, Sarah Barrière, Eric Bertin, Arthur Kaladjian

**Affiliations:** 1 Psychiatry Department, Reims University Hospital, EPSM Marne, 51100 Reims, France; 2 Cognition Health and Society Laboratory (EA 6291), Université de Reims Champagne-Ardenne, 51100 Reims, France; 3 Reims Champagne-Ardenne University, Reims, France; 4 Champagne Ardenne Specialized Center in Obesity, University Hospital Center, 51100 Reims, France

**Keywords:** Obesity, Emotions, Thoughts, (Lack of) perseverance, Urgency, Bariatric surgery

## Abstract

**Background:**

Even if bariatric surgery is considered the most effective therapeutic approach, it is not equally successful among individuals suffering from severe obesity and candidates for this weight loss surgery. Among the factors that influence postsurgical outcomes, eating behaviors styles are known to play a key role in relapses. The aim of our study was to assess eating behaviors styles and several modulating psychopathological factors in patients suffering from severe obesity.

**Methods:**

Patients seeking bariatric surgery (N = 127) completed a set of standardized tools assessing eating behaviors (Dutch Eating Behavior Questionnaire), comorbid psychiatric conditions (Mini International Neuropsychiatric Interview), depression, and anxiety scores (Beck Depression Inventory, State-Trait Anxiety Inventory), and impulsivity scores (UPPS-P Impulsive Behavior Scale).

**Results:**

We detected significant correlations between DEBQ Emotional Eating (EmoE) and depression, state and trait anxiety, and all dimensions of impulsivity. Significant correlations were also present between DEBQ External Eating (ExtE) and depression, state and trait anxiety and UPPS-P positive urgency, lack of perseverance and sensation seeking. Regression analyses identified sex (female), trait anxiety, and lack of perseverance as explanatory factors for EmoE, and depression severity score and positive urgency for ExtE.

**Conclusions:**

EmoE might be a means of dealing with negative emotions and/or intrusive thoughts, while ExtE might result from a mechanism associated with depression. These results should help to improve patients’ outcomes by defining specific therapeutic targets in psychological interventions.

**Plain English summary:**

After bariatric surgery, some patients regain weight. This is likely due to various factors, including a return of maladaptive eating styles, such as emotional eating (which occurs as a response to negative emotions, like depression, anxiety, anger, sadness, and discouragement), external eating (which refers to the tendency to eat in response to positive external cues, regardless of internal signals of hunger and satiety), and restraint eating (implying to make efforts to develop and maintain strategies to control calories intake, associated with weight loss after lifestyle intervention). Our goal in this research project was to explore associated factors (particularly depression, anxiety, and impulsivity) to these eating styles in patients suffering from obesity prior to bariatric surgery. Individuals seeking bariatric surgery were asked questions about their eating styles and their levels of depression, anxiety, and impulsivity using standardized questionnaires. We found that emotional eating might be a means of dealing with negative emotions and/or intrusive thoughts (e.g. about food or body dissatisfaction), while external eating might result from a mechanism associated with depression. We detected no association between restraint eating and any of the dimensions of impulsivity, nor depression and anxiety. Therapies aimed at improving patients’ abilities to regulate negative affects seem promising among subjects suffering from obesity and those seeking bariatric surgery. If well learned, these therapies might also help them to maintain weight loss after surgery by limiting maladaptive eating styles.

## Background

Obesity is now recognized as having reached an epidemic level worldwide and is associated with many serious diseases in developed countries. In 2014, more than 1.9 billion adult individuals were overweight, and 600 million were obese (defined as those having a body mass index (BMI) greater than or equal to 30 kg/m^2^) [1]. Individuals with BMI ≥ 35 kg/m^2^ (grade 2 obesity) with at least one comorbid condition and those with BMI ≥ 40 kg/m^2^ (grade 3 obesity) can benefit from bariatric surgery, which is considered the most effective weight loss treatment [2]. Indeed, an average of 50% of excess weight may be lost in the first few years after bariatric surgery, which secondary improves comorbidities like diabetes mellitus and cardiovascular events [3–6]. However, bariatric surgery is not equally effective in all patients, and many factors influencing long-term postsurgical outcomes are still to be understood in this population in which high rates of psychopathology have been reported [7].

Among these factors, eating behaviors styles may play a key role in success after weight loss surgery [8]. Indeed, Bryant et al. [9] suggested that the level of disinhibition, restraint, hunger, emotional eating, and uncontrolled eating could influence an individual’s ability to successfully regulate their energy intake post-surgery. High levels of pre-surgical emotional eating were associated with poor weight loss 1 year after surgery [10], and a higher score on emotional eating in women suffering from obesity who underwent a gastric bypass was associated with an insufficient weight loss 2 years after surgery [11]. It was also suggested, using the Three-Factor Eating Questionnaire (TFEQ), that a higher vulnerability to various internal (i.e. negative emotions) and external eating cues (i.e. scent and sight of food, and social events) shortly after surgery was the main predictor of less successful short- and long-term weight loss [8]. Moreover, as suggested in the systematic review by Athanasiadis et al. [12], emotional eating, food urges, binge eating, and loss of control/disinhibition while eating have all been positively associated with weight regain after bariatric surgery.

In a clinical context, the Dutch Eating Behavior Questionnaire (DEBQ), like the TFEQ, has been largely used to assess eating styles [13]. This questionnaire arises from the psychosomatic, the externality, and the restraint theories related to three kinds of eating styles: Emotional Eating (EmoE), External Eating (ExtE), and Restraint Eating (RE) [14]. In EmoE, eating occurs as a response to negative emotions, such as depression, anxiety, anger, sadness, and discouragement [15]. ExtE refers to the tendency to eat in response to positive external cues, such as the sight or the smell of food, regardless of internal signals of hunger and satiety [14]. RE implies making efforts to develop and maintain strategies to control calorie intake in order to obtain weight loss [16]. Psychological factors have been shown to contribute to those eating behavior styles among bariatric surgery candidates. Positive correlations have been reported between the severity of depression and both EmoE and ExtE; while a negative correlation has been shown between severity of depression and RE [17, 18]. Furthermore, EmoE, and ExtE were shown to be correlated with anxiety scores [18].

Impulsivity, a complex and multifaceted construct [19], is also important to consider. Authors have reported that EmoE, ExtE, and RE were associated with increased impulsivity in overweight and individuals suffering from obesity [20], even if such an association seems to be limited to responsiveness to rewards and reduced response inhibition [21]. Moreover, impulsivity may be associated with less weight loss after surgery, even if it is argued that it may be specific to state impulsivity [22]. As the direct effect of trait impulsivity on weight loss is not confirmed, this might be due to an indirect effect through eating behaviors (eating behaviors might be viewed as mediation variables).

Based on these previous theoretical considerations and empirical results, the aim of our work was to assess if depression, anxiety, and impulsivity traits represent important contributors to eating behaviors styles in patients suffering from obesity seeking bariatric surgery. We expected to find strong links between depression, anxiety, and EmoE or ExtE. We also expected to find a relationship between impulsivity and EmoE or ExtE, given their strong co-occurrence [23]. For RE, expectations are more uncertain given the findings noted above.

## Materials and methods

### Participants

A total of 155 participants, all candidates for bariatric surgery, were recruited in a ward specialized in severe obesity in Reims (France) from October 2017 to January 2019. From this pool we filtered out scales with missing responses (n = 25) and as impulsivity is a construct affected by addiction, participants with present or past drug or alcohol abuse or dependence were not included (n = 3). The final sample consisted of 127 participants. The participants were told that the study would enable a better understanding of the difficulties encountered by bariatric surgery candidates, and that there was no financial compensation. Although the study was proposed before the approval process for the surgery, they were also told that their results would not impact their treatment and the selection process. The inclusion criteria were obesity grade 2 (BMI 35.0–39.9 kg/m^2^) plus at least one obesity-related comorbidity or obesity grade 3 (BMI ≥ 40.0 kg/m^2^), and all bariatric surgery candidates were without an actual anorexia nervosa, bulimia or binge-eating disorder as assessed by a structured interview. Patients had to be French-speaking and to be 18–65 years old. All study procedures were reviewed and approved by the local Institutional Review Board (Dossier IRB 2016-12). The study was carried out according to the Helsinki Declaration [24], and every patient included in the study provided written informed consent.

#### Assessments

For all participants, we assessed sex, age, and BMI (kg/m^2^; calculated from the measured weight and height at data collection). Patients completed a set of standardized tools assessing eating behaviors (Dutch Eating Behavior Questionnaire), comorbid psychiatric conditions (Mini International Neuropsychiatric Interview), depression, and anxiety scores (Beck Depression Inventory, State-Trait Anxiety Inventory), and impulsivity scores (UPPS-P Impulsive Behavior Scale).

##### Eating behaviors styles

The Dutch Eating Behavior Questionnaire (DEBQ), a self-report scale, was administered to assess Emotional Eating (eating as a response to negative emotions), External Eating (eating in response to positive external cues), and Restraint Eating (efforts to develop and maintain strategies to control calorie intake) [13, 25]. The DEBQ consists of 33 items answered on a 5-point Likert scale (ranging from “never” to “very often”). Thirteen items evaluate EmoE, and 10 items evaluate ExtE and RE. The Cronbach’s alpha values were 0.959 for EmoE, 0.748 for ExtE, and 0.855 for RE.

##### Comorbid psychiatric conditions

We used the Mini International Neuropsychiatric Interview (a structured interview) (French Version 5.0.0) (MINI) to investigate past and current psychiatric disorders, including alcohol abuse and dependence, drug abuse and dependence or anxiety disorders. The MINI contains 130 questions and screens 16 axis I DSM-IV disorders and one personality disorder (i.e., antisocial personality disorder).

##### Depression severity and anxiety

The severity of depression was assessed using the short version of the Beck Depression Inventory, which is a widely used self-report scale (BDI-13) [26, 27]. The total score is obtained by adding the scores of 13 items and ranges from 0 to 39, with higher scores indicating greater depressive symptoms. Cronbach’s alpha was 0.837. Anxiety was evaluated with the State-Trait Anxiety Inventory (STAI), a self-report scale [28]. Trait anxiety refers to how dispositionally anxious a person is across time and situations; state anxiety refers to how anxious a person is feeling at a particular moment. This inventory is a 40-item scale (20 items evaluate trait anxiety, and 20 items evaluate state anxiety), using a 4-point Likert scale for each item. The Cronbach’s alpha value was 0.945 for the state scale and 0.933 for the trait scale.

##### Impulsivity

We used the UPPS-P Impulsive Behavior Scale [29] to assess the facets of impulsivity, i.e., Negative Urgency, Positive Urgency, (lack of) Premeditation, (lack of) Perseverance, and Sensation Seeking. This shortened version (20 items) has 4 items per scale, and each item is responded to on a 4-point Likert-type scale. This self-report scale has received endorsement from the National Institutes of Health’s (NIH) PhenX Toolkit as the recommended self-report measure of impulsigenic traits [30]. The Cronbach’s alpha values in our sample were 0.796, 0.703, 0.754, 0.738, and 0.623, respectively.

### Data analytic plan

All variables were screened for extreme outliers using box plot analyses. Descriptive (degree of skewness and kurtosis) and graphical methods were used to examine whether data were normally distributed. The relationships between the eating style scores, clinical characteristics, and impulsivity dimensions were examined using Pearson’s correlation, and separate hierarchical multiple regressions were performed to determine which variables predicted each of the eating behaviors styles as measured by the DEBQ subscales. All variables were considered clinically relevant and selected for all regression models. We constructed three blocks: (1) demographic information (i.e., age, sex), (2) affective symptoms (i.e., anxiety and depression), and (3) impulsivity dimensions (i.e., UPPS-P). Because DEBQ scores are known to be influenced by age and sex, these variables were forced as covariates in the first step. Then, anxiety (trait and state scores) and depression severity were entered in the second step, and all the impulsivity scores were entered in the third step. A forward selection was performed in steps 2 and 3. To test for multicollinearity, we examined the variance inflation factor (VIF) and the tolerance values (1/VIF). The tolerance ranged from 0.819 to 0.957, indicating that there was no issue [31]. All the statistical analyses were performed with SPSS® software, version 24.0 (SPSS, Inc., Chicago, IL, USA) and *p *values < 0.05 were regarded as statistically significant.

## Results

### Clinical characteristics

Most participants were female (N = 88, 69.29%) and ranged from 20 to 61 years old, with a mean age of 41.27 ± 11.20 years old (females 39.19 ± 11.12 years old; males 45.95 ± 10.01 years old). The mean BMI was 45.65 ± 6.72 kg/m^2^ (females 45.85 ± 6.86 kg/m^2^; males 45.22 ± 6.47 kg/m^2^).

Eating behaviors styles, depression, anxiety, and impulsivity scores are shown in Table 1 for the overall population and according to sex.

**Table 1:** Descriptive data for eating behaviors styles, depression, anxiety, and impulsivity scores in the overall population, in females, and in males

	Overall population (N = 127)		Females (N = 88)		Males (N = 39)	
	Mean (Min–Max)	SD	Mean (Min–Max)	SD	Mean (Min–Max)	SD
Emotional eating	2.38 (0.85–5.00)	1.04	2.59 (0.85–5.00)	1.06	1.91 (0.92–3.69)	.85
External eating	2.57 (1.10–4.20)	.62	2.57 (1.10–4.20)	.59	2.56 (1.20–3.90)	.70
Restraint eating	2.77 (1.00–4.60)	.77	2.78 (1.30–4.60)	.78	2.74 (1.00–4.60)	.76
BDI^a^	9.55 (0–31)	6.52	10.26 (0–27)	6.70	7.95 (0–31)	5.87
STAI-A^b^	33.76 (20–76)	12.08	33.82 (20–76)	12.00	33.64 (20–71)	12.40
STAI-B^c^	37.99 (20–70)	11.76	30.09 (20–70)	11.96	35.51 (20–70)	11.05
UPPS negative urgency^d^	8.53 (4–15)	2.94	8.56 (4–15)	2.94	8.46 (4–15)	2.98
UPPS positive urgency^d^	9.46 (4–16)	2.45	9.57 (5–15)	2.37	9.21 (4–16)	2.64
UPPS lack of premeditation^d^	6.62 (4–11)	2.04	6.69 (4–11)	2.12	6.46 (4–10)	1.88
UPPS lack of perseverance^d^	6.06 (4–13)	2.07	6.28 (4–13)	2.16	5.56 (4–9)	1.77
UPPS sensation seeking^d^	8.49 (4–15)	2.37	8.49 (4–14)	2.34	8.49 (4–15)	2.66

^a^BDI = Beck Depression Inventory, ^b^STAI-A = State-Trait Anxiety Inventory (State), ^c^STAI-B = State-Trait Anxiety Inventory (Trait), ^d^UPPS = UPPS-P Impulsive Behavior Scale. Min = Minimum, Max = Maximum

### Links between clinical characteristics

Among the eating styles scores, EmoE was significantly correlated with ExtE, depression and anxiety scores, and all dimensions of impulsivity (Table 2). ExtE was significantly correlated with EmoE, depression and anxiety scores, positive urgency, lack of perseverance, and sensation seeking. RE was not correlated with any variable (Table 2). The male participants were older than the females, and the females presented more emotional eating than the males (Table 2).

**Table 2:** Bivariate correlations made in the whole sample between eating behaviors styles scores, clinical characteristics, and impulsivity dimensions scores

	1	2	3	4	5	6	7	8	9	10	11	12
1. Emotional Eating	–	–	–	–	–	–	–	–	–	–	–	
2. External Eating	.554**	–	–	–	–	–	–	–	–	–	–	
3. Restraint Eating	− .002	− .121	–	–	–	–	–	–	–	–	–	
4. Age	− .103	− .121	.038	–	–	–	–	–	–	–	–	
5. BDI^a^	.401**	.272**	.026	.258**	–	–	–	–	–	–	–	
6. STAI-A^b^	.364**	.206*	.049	− .194*	.664**	–	–	–	–	–	–	
7. STAI-B^c^	.407**	.248**	− .049	− .185*	.743**	.790**	–	–	–	–	–	
8. UPPS-P negative urgency^d^	.256**	.146	.015	.041	.418**	.468**	.517**	–	–	–	–	
9. UPPS-P positive ^urgencyd^	.201*	.219*	.013	.031	.227*	.163	.229**	.650**	–	–	–	
10. UPPS-P Lack of premeditation^d^	.200*	.088	− .117	.052	.207*	.288**	.349**	.299**	.162	–	–	
11. UPPS-P Lack of perseverance^d^	.382**	.220*	.005	− .042	.334**	.320**	.389**	.252**	.232**	.495**	–	
12. UPPS-P sensation seeking^d^	.204*	.236**	.008	− .004	.306**	.259**	.255**	.451**	.567**	.078	.134	
13 Gender^e^	− .304**	− .004	− .025	.279**	− .164	− .007	− .141	− .015	− .069	− .053	− .161	.000

^a^BDI = Beck Depression Inventory

^b^STAI-A = State-Trait Anxiety Inventory (State)

^c^STAI-B = State-Trait Anxiety Inventory (Trait)

^d^UPPS-P = UPPS-P Impulsive Behavior Scale

^e^Sex (0: females; 1: males)

^*^p < 0.05; **p < 0.01

### Relative contributions of depression, anxiety, and impulsiveness variables to eating behaviors styles scores

The first regression analysis, with EmoE as the dependent variable, showed a significant model in all three steps (F (2,124) = 6.34, *p* = 0.002 in step 1, including sex and age; F (3,123) = 12.17, *p* < 0.001 in step 2, including symptoms of anxiety trait; and F (4,122) = 11.54, *p* < 0.001 in step 3, including UPPS lack of perseverance). R square change was 9.3% in the first step (*p* = 0.002), 13.6% in the second step (*p* < 0.001), and 4.6% in the third step (*p* = 0.006) (Table 3).

**Table 3:** Hierarchical linear regressions of anxiety, depression severity, and impulsivity traits in relation to eating behaviors styles in the whole sample

	Emotional Eating Model: R = .52 Adjusted R^2^ = .25			External Eating Model: R = .33 Adjusted R^2^ = .08	
	Bêta	*p*		Bêta	*p*
Sex	− 0.233	0.005	Sex	0.069	0.440
Age	0.026	0.755	Age	− 0.089	0.334
STAI-B^a^	0.287	0.001	BDI^b^	0.221	0.017
UPPS^c^ lack of perseverance	0.234	0.006	UPPS^c^ positive urgency	0.177	0.048

This table depicts results from significant final steps of regression analyses (i.e., for EmoE and ExtE)

^a^STAI-B = State-Trait Anxiety Inventory (Trait), ^b^BDI = Beck Depression Inventory, ^c^UPPS = UPPS Impulsive Behavior Scale

The second regression analysis, with ExtE as the dependent variable, showed a significant model in the two last steps (F (2,124) = 0.984, *p* = 0.377 in step 1, including sex and age; F (3,123) = 3.573, *p* = 0.016 in step 2, including depression severity score; and F (4,122) = 3.746, *p* = 0.007 in step 3, including UPPS positive urgency). R square change was 1.6% in the first step (*p* = 0.377), 6.5% in step 2 (*p* = 0.004), and 2.9% in the third step (*p* = 0.048) (Table 3).

The third regression analysis, with RE as the dependent variable, showed a non-significant model (F (2,124) = 0.172, *p* = 0 0.842). No variable contributed significantly to the prediction of RE.

## Discussion

The current study aimed to examine the potential contributions of depression, anxiety, and the different facets of impulsivity to each of the three eating behaviors styles assessed by the DEBQ in patients presenting for bariatric surgery. We have found significant positive correlations between EmoE, and depression, state and trait anxiety, and all dimensions of impulsivity. Moreover, we have identified that sex (female), trait anxiety, and lack of perseverance were explanatory factors for EmoE. Significant positive correlations have also been found between ExtE, and depression, state and trait anxiety and UPPS-P positive urgency, lack of perseverance and sensation seeking. Depression severity score and positive urgency were explanatory factors for ExtE. No variable contributed significantly to the prediction of RE. In addition to confirming previous results regarding EmoE, ExtE, and depression, our study allows us to deepen our knowledge on the contributions of depression, anxiety, and impulsivity dimensions to different eating styles in this population of bariatric surgery.

Our results showed that females were at a higher risk than males for developing EmoE. This finding is consistent with the findings of Gade et al. [32] among patients suffering from obesity before gastric bypass surgery. Trait anxiety also appeared as an explanatory factor for EmoE. However, EmoE due to anxiety symptoms has been demonstrated as less associated with psychopathological phenomena than those due to depression [33]. Thus, the emotional dysregulation known to occur in depression and not negative affects by themselves (i.e. emotional responses to real or perceived imminent threat or anticipation of future threat) might be considered as the drivers of EmoE [33, 34]. Lack of perseverance was the only facet of impulsivity found as an explanatory factor in EmoE (also confirmed by cross-validation of our regression model). This dimension of impulsivity is defined as the difficulty in staying focused on a task because of the intrusion into the memory of thoughts and memories previously useful but irrelevant to the current task [35–37]. Hence, one of the mechanisms of EmoE would be the existence of intrusive thoughts, although our study did not allow us to determine whether the topic of these thoughts were food, weight, body dissatisfaction, or manifestations of post-traumatic stress disorder, as has been observed in patients suffering from bulimia or among bariatric surgery candidates [38–41]. Canale et al. [42] have proposed a disruptive role of emotions on the resistance to proactive interference process. Thus, intense emotions would lead to strong intrusive thoughts and then food intake to try to cope with these. This hypothesis is supported by the existence of increased activity in the orbitofrontal cortex in participants who have received a negative mood induction and have been exposed to images of appetizing foods, reflecting a rise in the value of reward food cues in case of negative affects [43]. In summary, this brings to the fore the importance of intrusive thoughts in EmoE and suggests that the lack of inhibition of mental images/thoughts could be responsible for a loss of control of food intake. Eating would thus be an ineffective and inappropriate means to cope with emotions, which, by force of repetition and mental elaboration, could be automatized and self-sustained.

ExtE remains associated with unhealthy food intake [20, 44, 45] and a serious increase in BMI [46, 47], even if it is to a lesser extent than EmoE. Our analysis adjusted for sex and age showed that depression severity and positive urgency predicted this eating style. Sevinçer et al. [17] suggested a positive correlation between the severity of depression and ExtE among bariatric surgery candidates. In a population of adults from the community, general practice, and specialized mental health care, Paans et al. [48] found that depression and unhealthy eating styles contributed independently to poorer dietary quality and higher intake of sweet foods and fast-food/savory snacks [48]. These authors also found that the association between depression and higher intake of snack/fast-food was explained by ExtE, suggesting that ExtE cues may be an important mechanism linking depression and obesity. Positive urgency, the tendency to act rashly in response to positive emotions, appears to be less relevant for dysregulated eating [49].

Regarding RE, no association was found with any of the dimensions of impulsivity, nor depression and anxiety. Available data on the subject are still heterogeneous [20, 21, 50] and remain to be qualified. However, using TFEQ, Hindle et al. [51] showed that patients with higher cognitive restriction before surgery perceived less reduction in hunger after surgery; this eating behavior style thus requires further studies addressing its correlates. Given differences between the scales used to identify RE, several types of restrictive eating behaviors could be distinguished, corresponding to different personality profiles, different impulsivity traits, and different levels of success in terms of weight loss [21]. In DEBQ, RE corresponds to the existence of behavioral strategies used to control energy inputs, followed by success or not [13, 52]. RE might thus include a broad range of individual profiles. This might explain why results are mixed in the literature and why our study does not find a correlation with the explored dimensions of impulsivity. More generally, based on our results, we can assume that food restriction alone, and as defined by the DEBQ, does not necessarily mean a high level of impulsivity and high likelihood of weight regain, as long as some personality traits are not associated with. On the other hand, our study population was likely to display a particular type of DEBQ-RE: their restriction goals were a priori defeated. Moreover, the evaluation mode (by self-reports) is a potential source of response bias (self-complacency or self-handicap bias) because individuals may believe that they had interest in indicating that they have no more implemented restriction strategies even if some of them might present a restraint eating style (implying that these alone have not been enough, and that surgery is therefore necessary). The exclusion of individuals with eating disorders from our study population may also explain conflicting findings compared to other studies.

This study has several limitations. The first is that the population was limited to only candidates for bariatric surgery, which limits the generalizability of the findings. For instance, moderate obesity was not present in the current sample, in addition to other exclusion criteria, including anorexia, bulimia, binge-eating disorder, and an addiction to a psychoactive substance. Our sample was, therefore, only representative of this specific situation. However, there may be an advantage in having targeted this population because eating disorders are themselves very strongly associated with impulsivity, with heterogeneous results according to their type. Because explanatory models are probably very different based on the presence of an eating disorder or not, our results make it possible to study—in a more specific way—the mechanisms of each eating behavior style in individuals without eating disorders. Our study also makes it possible to focus on people seeking bariatric surgery, to adapt their care, and so, to minimize the risk of relapse. Future studies could be conducted to investigate the effect of focused therapies on relapse in these patients. A second limitation comes from the unbalanced sex ratio: 69.3% of participants were female. And lastly, we assessed depression, anxiety, impulsivity, and eating behaviors styles through self-reports, which are subjects to possible bias. However, the validity of these questionnaires has been well supported in previous studies, and our reliability indices were satisfactory.

## Conclusions

The results of this study suggest that in individuals suffering from obesity seeking bariatric surgery, trait anxiety and a lack of resistance to proactive interference signal risk of EmoE, while depression severity and the tendency to act rashly in response to positive emotions signal risk of ExtE. Therapies targeting abilities to regulate negative affects and impulsivity look promising and require further studies.

## Data Availability

The qualitative datasets (i.e., transcripts, coding, and themes) used and/or analyzed during the current study are available from the corresponding author upon reasonable request.

## Abbreviations

BDI: Beck Depression Inventory
BMI: Body mass index
DEBQ: Dutch Eating Behavior Questionnaire
EmoE: Emotional Eating
ExtE: External Eating
MINI: Mini International Neuropsychiatric Interview
RE: Restraint Eating
STAI: State-Trait Anxiety Inventory
STAI-A: State-Trait Anxiety Inventory (State)
STAI-B: State-Trait Anxiety Inventory (Trait)
TFEQ: Three-Factor Eating Questionnaire
UPPS: UPPS Impulsive Behavior Scale
